# Wearable Ion Sensors for the Detection of Sweat Ions Fabricated by Heat-Transfer Printing

**DOI:** 10.1021/acssensors.3c01027

**Published:** 2023-06-15

**Authors:** Isao Shitanda, Naoki Muramatsu, Rio Kimura, Nanami Takahashi, Kazuki Watanabe, Hiroyuki Matsui, Noya Loew, Masahiro Motosuke, Takahiro Mukaimoto, Momoko Kobayashi, Taketo Mitsuhara, Yamato Sugita, Kensuke Matsuo, Shinya Yanagita, Tatsunori Suzuki, Hikari Watanabe, Masayuki Itagaki

**Affiliations:** † Department of Pure and Applied Chemistry, Faculty of Science and Technology, Tokyo University of Science, 2641 Yamazaki, Noda 278-8510, Chiba, Japan; ‡ Research Institute for Science and Technology, Tokyo University of Science, 2641 Yamazaki, Noda 278-8510, Chiba, Japan; § Research Center for Organic Electronics (ROEL), Yamagata University, 4-3-16 Jonan, Yonezawa 992-8510, Yamagata, Japan; ∥ Department of Mechanical Engineering, Faculty of Engineering, Tokyo University of Science, 6-3-1 Niijuku, Katsushika-ku, Tokyo 125-8585, Japan; ⊥ Institute of Arts and Sciences, Tokyo University of Science, 2641 Yamazaki, Noda 278-8510, Chiba, Japan; # Department of Pharmacy, Faculty of Pharmaceutical Sciences, Tokyo University of Science, 2641 Yamazaki, Noda 278-8510, Chiba, Japan; ∇ Department of Globe Fire Science and Technology, Faculty of Science and Technology, Tokyo University of Science, 2641 Yamazaki, Noda 278-8510, Chiba, Japan

**Keywords:** wearable, ion sensor, screen printing, heat-transfer printing, on-body testing

## Abstract

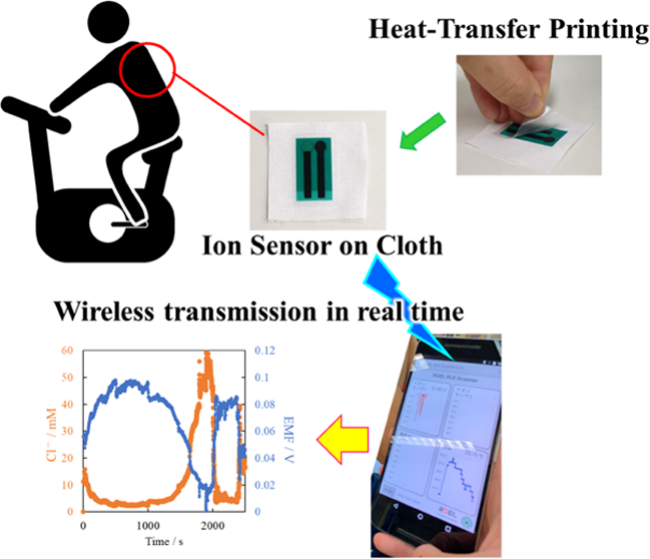

Wearable ion sensors for the real-time monitoring of sweat biomarkers have recently attracted increasing research attention. Here, we fabricated a novel chloride ion sensor for real-time sweat monitoring. The printed sensor was heat-transferred onto nonwoven cloth, allowing for easy attachment to various types of clothing, including simple garments. Additionally, the cloth prevents contact between the skin and the sensor and acts as a flow path. The change in the electromotive force of the chloride ion sensor was −59.5 mTV/log *C*_Cl^–^_. In addition, the sensor showed a good linear relationship with the concentration range of chloride ions in human sweat. Moreover, the sensor displayed a Nernst response, confirming no changes in the film composition due to heat transfer. Finally, the fabricated ion sensors were applied to the skin of a human volunteer subjected to an exercise test. In addition, a wireless transmitter was combined with the sensor to wirelessly monitor ions in sweat. The sensors showed significant responses to both sweat perspiration and exercise intensity. Thus, our research demonstrates the potential of using wearable ion sensors for the real-time monitoring of sweat biomarkers, which could significantly impact the development of personalized healthcare.

Devices that apply various sensors have been developed for health checks and performance evaluations during exercise. These sensors can assess the health status of a subject by detecting vital signs. Many wearable sensors can cause stress on the subject, such as by the pressure applied when measuring heart rate or by drawing blood to measure blood glucose levels. To solve this problem, the development of wearable devices has attracted increased research attention in recent years.^1−5^ This paper describes the development of a wearable sensor for monitoring various health indicators. Wearable sensors are devices worn on the body to measure vital signs such as heart rate, respiration rate, body temperature, blood pressure, and blood glucose levels. The advantages of wearable devices include the ability to monitor vital signs continuously during daily life and exercise without the need to collect blood. As the public is becoming increasingly health-conscious, attention to wearable devices that can continuously monitor vital signs is gaining increasing attention. For instance, physical sensors that can be applied to the skin to monitor muscle, heart, and brain activity in real time have been developed.^6,7^ However, there are few reports of the development of chemical sensors and biosensors that can be used to measure sugar and lactate levels in a noninvasive manner without damaging the skin. These types of wearable chemical sensors or biosensors are expected to provide more detailed health monitoring than physical sensors. Therefore, the development of chemical sensors and biosensors plays a major role in the progression of this field.

One method for the assessment of health is a component analysis of body fluids. Various health parameters are contained in human body fluids, such as sweat, saliva, and tears, and are correlated with those contained in the blood. Therefore, it is possible to monitor health status by monitoring various parameters contained in body fluid without collecting blood. Wang et al. developed a wearable sensor that uses tattoo paper as a substrate to monitor lactate concentration in sweat.^8^ Coyle and colleagues developed a wearable sensor that uses a textile substrate to monitor sweat pH, sodium concentration, sweat rate, and conductivity.^9^ Mitsubayashi et al. developed a contact lens-type biosensor that measures the glucose concentration in tears.^10^ Our group has previously reported the development of wearable devices that use biofuel cells, such as a urine sugar sensor that can be incorporated into diapers,^11^ and a wearable power source that generates electricity when wetted by perspiration.^12^

Wearable devices can monitor the chloride concentrations in sweat as an indicator of health.^13−19^ Because chloride is the most abundant electrolyte in human sweat, measuring its concentration provides an excellent indicator of the body’s electrolyte balance and is also useful for the diagnosis and prevention of heat stroke, such as hyponatremia.^20−23^ Consequently, various types of wearable sensors have been reported,^24−35^ such as a wearable ion sensor with a tattoo paper substrate reported by Wang et al.^24,35^ However, monitoring chloride ions in sweat by wearing a sensor using tattoo paper as a substrate is uncomfortable due to skin irritation caused by sweat. On the other hand, when a sensor is formed directly into a wearable textile, the resistance increases and the accuracy decreases because of surface irregularities and lack of ink penetration into the textile. In this study, we developed a new wearable sensor that can quantify chloride ions in sweat using a technique called transfer printing. Transfer printing is used to print on curved surfaces that cannot be printed directly, such as on fragile objects or on the skin. The methodology of transfer printing involves printing onto a substrate, which is then transferred to the required nonplanar object by either heat or water. Sensors are screen-printed onto textiles via the transfer printing technique using polyester films. A release agent peels off the adhered layer by applying heat to add the sensor to the cloth. Using resistant ink and an overcoat as the protective layer, the sensor is protected from heat and pressure during transfer by sandwiching the sensor part between two resist layers. The process of transferring sensors to rough paper or textiles is made possible by high-precision printing devices. Additionally, the sensor can be transferred to fiber substrates so that they can be incorporated into textiles such as T-shirts, wristbands, and insoles so that health indicators can be measured simply by wearing them.

While the main focus of this study was on chloride ion sensors, sodium, potassium, and ammonium ion sensors were also fabricated.

## Experimental Section

### Reagents

Chloride ionophore I, tridodecyl(methyl)ammonium chloride (TDDMACl), poly(vinyl chloride) (PVC), tetrahydrofuran (THF), poly(vinyl butyral) BUTVAR B-98 (PVB), and Pluronic F-127 were purchased from Sigma-Aldrich. *o*-Nitrophenyl octyl ether (*o*-NPOE) was purchased from Tokyo Chemical Industry Co., Ltd. Sodium chloride (NaCl), ammonium sulfide ((NH_4_)_2_SO_4_), potassium nitrate (KNO_3_), magnesium sulfide (MgSO_4_), glucose, and urea were purchased from Wako Pure Chemical Industries, Ltd. Conductive carbon ink, silver ink, and resist ink were purchased from Jujo Chemicals. The printing screen patterns were designed using AutoCAD (Autodesk) and fabricated by Mitani Micronics Co., Ltd. The poly(ethylene terephthalate) (PET) substrate, overcoat ink, and adhesive were obtained from Japan Polymer Co., Ltd. Artificial sweat contains NaCl, (NH_4_)_2_SO_4_, KNO_3_, MgSO_4_, glucose, and urea at physiological concentrations and is composed of various interfering electrolytes. The superabsorbent fibers (SAF) used as sweat channels were provided by Oji Holdings.

### Equipment

Screen printing was performed using an LS-150TV (Newlong Seimitsu Kogyo Co., Ltd.), which is a semiautomatic screen printer. The electrochemical characteristics were evaluated using the potentiostat EmStat (PalmSens Co., Ltd.).

### Fabrication of the Electrode

**Figure 1:**
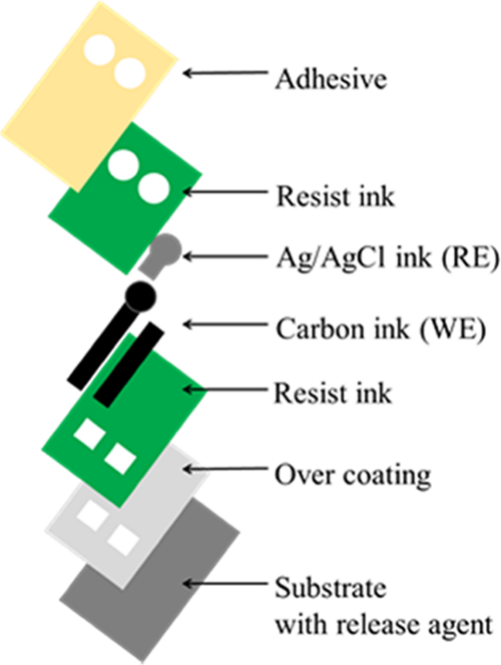
Schematic diagram of the printing layers.

A schematic diagram of the fabricated electrodes is shown in Figure 1. The electrodes were fabricated by printing and laminating overcoat ink, carbon ink, Ag/AgCl ink, resist ink, and adhesive, as shown in Figure 1, on a PET substrate treated with a release agent.

### Fabrication of Chloride Ion Sensor

For the chloride ion sensor, a mixture of 3.5 mg of chloride ionophore I, 51 mg of o-NPOE, 34 mg of PVC, and 2 mg of TDDMACl was dissolved in 1 mL of THF. Eight microliters of this mixture were dropped onto a carbon electrode and dried overnight to form a chloride ion-selective membrane.^36−38^ For the reference electrode membrane, a mixture of 120 mg of NaCl, 150 mg of PVB, 5 mg of Pluronic F-127, and 1 mL of methanol was prepared. Eight microliters of this mixture were dropped onto the Ag/AgCl electrode and dried overnight to form a film.^39−41^

### Heat-Transfer Printing

**Figure 2:**
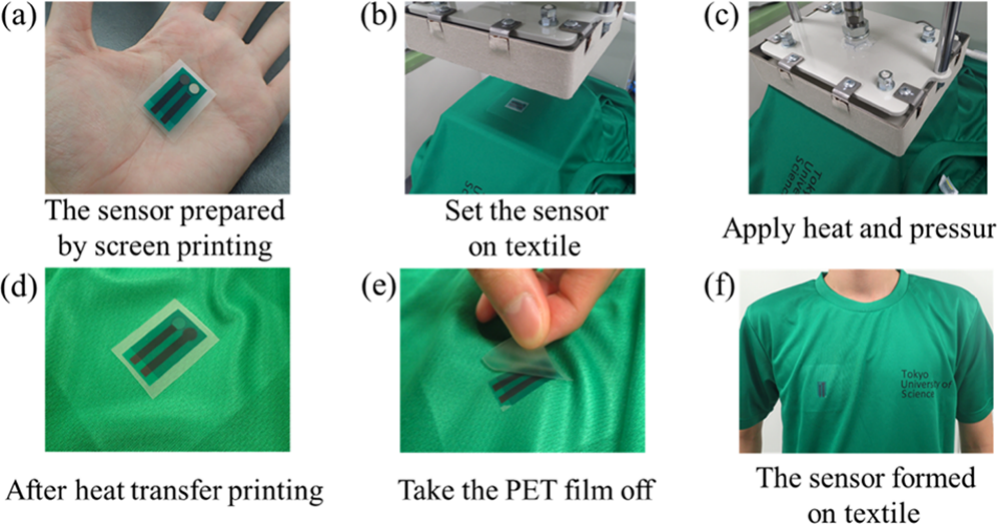
Heat-transfer process. (a) Screen-printed sensor on PET substrate. (b) Sensor on PET substrate placed on textile. (c) Application of heat and pressure. (d) Heat-transferred sensor before removal of PET substrate. (e) PET substrate is easily peeled off. (f) Sensor on textile.

The sensor was transferred onto textiles via heat-transfer printing using a Pony50 (Japan Polymerk Co., Ltd.) heat press. The fabricated sensor (Figure 2a) was placed on the textile (Figure 2b), and heat and pressure were applied using a heat press (Figure 2c). By applying a high temperature, the adhesive in contact with the cloth dissolves and the sensor is fixed on the textile (Figure 2d). Simultaneously, the release agent loses its adhesiveness when a high temperature is applied, separating the PET substrate from the sensor, leaving only the sensor on the textile (Figure 2e,f).

## Results and Discussion

### Ion Sensor Design

The ion sensor needed to be comfortable to wear (i.e., light weight, nonirritating, and soft); therefore, designing a textile-based sensor was an obvious choice as textiles are light weight, soft, and nonirritating when attached to the skin. The wicking effect of cloth is also an advantage as it contributes to spreading sweat evenly between the electrodes, establishing and maintaining electrical contact. However, the materials used to fabricate the sensor onto the cloth might be irritating when in direct contact with skin. Thus, the sensor was designed to be fabricated on the side of the cloth facing away from the skin during usage (Figure S1).

Screen-printing directly on textiles often leads to blurred edges on the printed pattern. This is avoided by using heat-transfer printing. Another advantage of this technique is that the sensor component layers are fabricated in reverse order (Figure 1). Thus, the ion-selective and reference electrode membranes, which are fabricated last by drop-casting, are the innermost layers of the completed sensor (Figure S1). With this design, sweat absorbed by the cloth quickly reaches the sensing area. Furthermore, only the cloth is in direct contact with the skin, avoiding possible irritation and improving comfort.

Finally, materials and sensing mechanisms were chosen carefully to avoid risking an allergic reaction during sensor usage. In particular, a chloride-selective-membrane electrode was chosen over an Ag/AgCl electrode for chloride sensing, because with the latter, there is a risk of leaking Ag^+^ ions provoking an allergic reaction in persons with metal allergies.

### Evaluation of the Electrode Performance

The individual electrodes were characterized by measuring the electromotive force (EMF) change between each electrode and a commercial Ag/AgCl/sat. KCl reference electrode in the presence of different concentrations of chloride ions. The ion-selective electrode or the fabricated reference electrode was immersed in a sodium chloride solution, the commercial reference electrode was immersed in a saturated potassium chloride solution, and the two solutions were connected by a salt bridge. The concentration of the sodium chloride solution was varied by an order of magnitude in the range of 0.1–100 mM, and the open-circuit potential was measured for 5 min each for the ion-selective electrode and 1 h each for the fabricated reference electrode.

**Figure 3:**
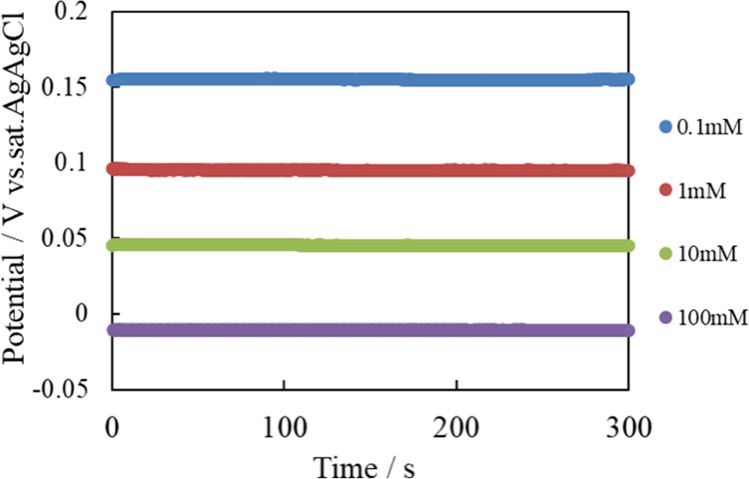
Open-circuit potential of chloride ion-selective electrode vs. commercial reference electrode in the presence of various chloride ion concentrations.

The potential of the chloride ion-selective electrode decreased with increasing chloride ion concentration (Figure 3). In Figure S2, the electric potential at 60 s after the start of measurement is plotted against the chloride ion concentration on a logarithmic scale. The relationship between the potential and logarithmic chloride ion concentration was linear, with a slope of −61.1 ± 1.8 mV/log *C*_Cl^–^_ (*n* = 3). Since the concentration of chloride ions in human sweat is 10–100 mM,^42^ we suggest that the fabricated chloride ion-selective electrode can be used to determine chloride ions in human sweat.

**Figure 4:**
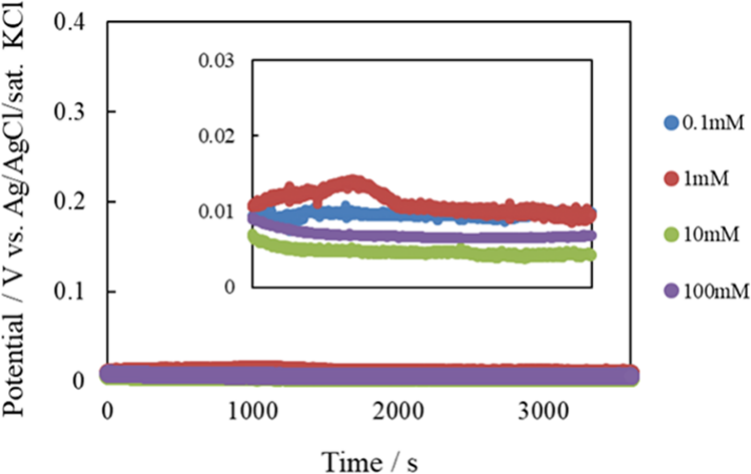
Open-circuit potential of fabricated reference electrode vs. commercial reference electrode in the presence of various sodium chloride concentrations.

The potential of the fabricated reference electrode was found to be independent of the chloride ion concentration (Figure 4). Therefore, the fabricated electrode is suitable as a reference electrode.

No attempts were made to physically straighten or stabilize the sensors during storage, handling, or measurement. Therefore, all responses shown here include possible errors due to slight temporary bending. The absence of unusual errors indicates that the sensors were reasonably flexible.

### Characterization of the Ion Sensor

The responses of the chloride ion sensors were evaluated by measuring the change in EMF between the ion-selective electrode and the fabricated reference electrode in the presence of different chloride ion concentrations in the range of 0.1–100 mM in artificial sweat.

**Figure 5:**
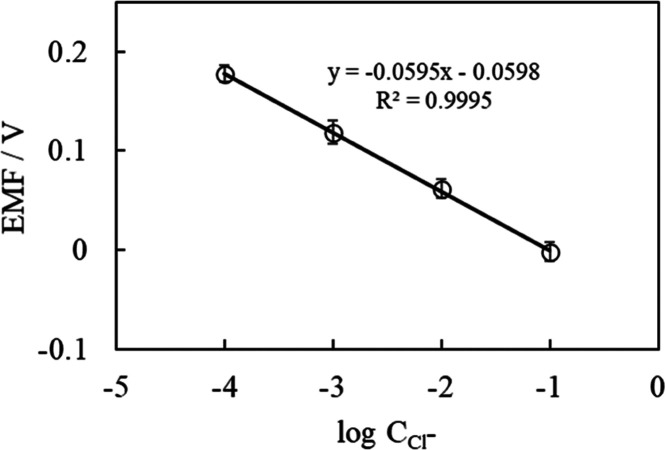
EMF of chloride sensor depending on chloride ion concentration in artificial sweat.

The responses were similar to those obtained while evaluating the individual electrode (Nernst response), with the potential depending on the chloride ion concentration. Figure 5 shows the results of plotting the electric potential 60 s after the start of the measurement against the concentration. After the transfer, the sensor showed potential changes according to the chloride concentration without interference from other biological species in artificial sweat. The graph was linear, with a slope of −59.5 ± 3.4 mV/log *C*_Cl^–^_ (*n* = 3). The lower detection limit was 1 × 10^–4.3^ M. Because the chloride ion concentration in human sweat is 10–100 mM,^42^ the chloride ion sensor formed on the textile substrate by transfer printing can be used to determine the chloride ion concentration in human sweat.

Corresponding characterizations of a similarly fabricated sodium, ammonium, and potassium ion sensor in artificial sweat showed a slope of +56 mV/log *C*_Na^+^_ (*n* = 3; Figure S3), +56.1 mV/log *C*_NH_4_^+^_ (*n* = 3; Figure S4), and +53.1 mV/log *C*_K^+^_ (*n* = 3; Figure S5), respectively. All sensors showed Nernstian behavior.

**Figure 6:**
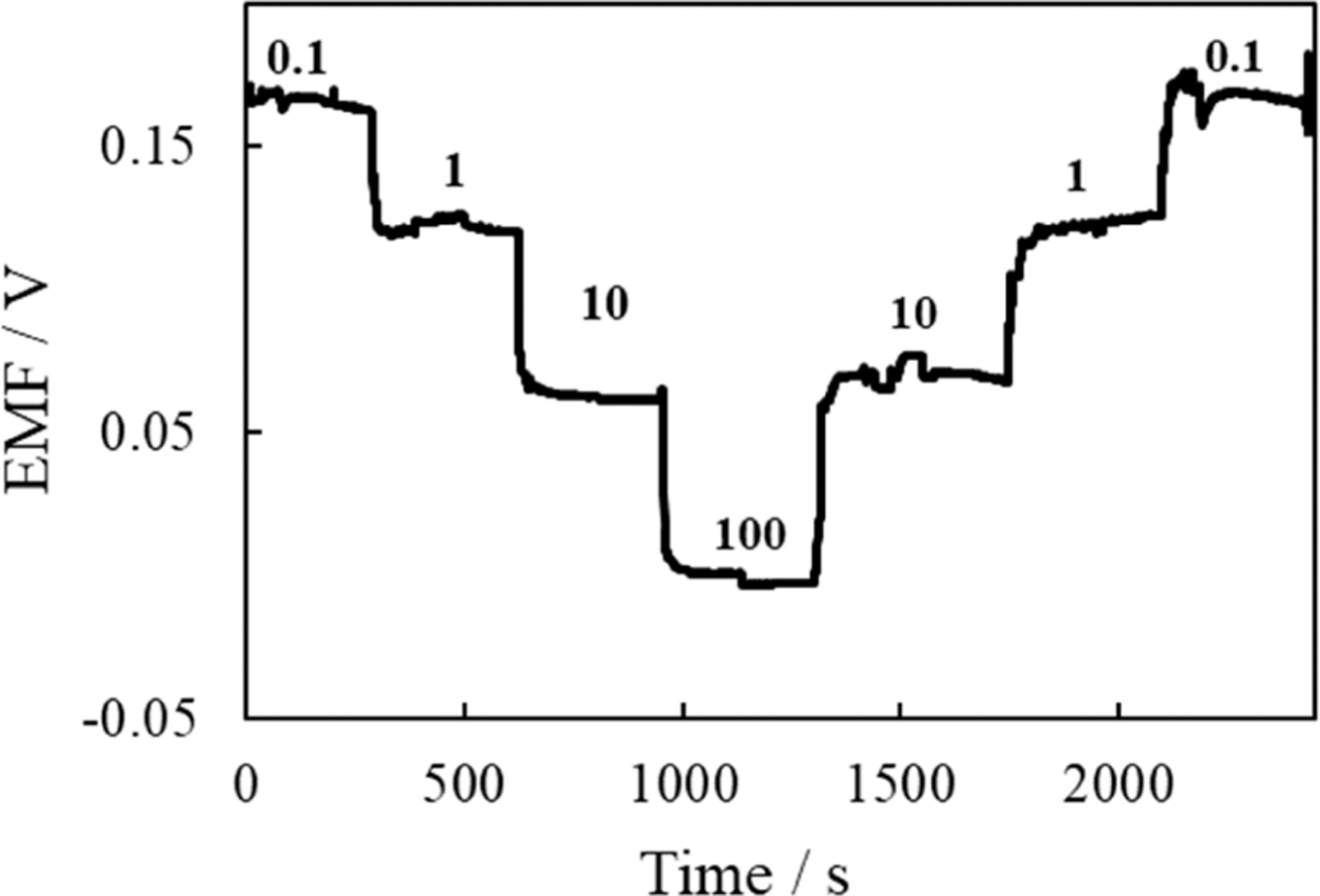
Continuous response of chloride ion sensor to stepwise changes in chloride ion concentration. The numbers above the line are respective chloride ion concentrations in mmol dm^–3^ (mM).

Next, the continuous sensor response to stepwise variations in the chloride ion concentration was evaluated. Figure 6 shows the response of the chloride ion sensor when the chloride ion concentration of the measurement solution was changed stepwise. The EMF of the sensor changed quickly and showed a stable potential in response to each chloride ion concentration change.

Furthermore, the response of the chloride ion sensor to a constant chloride ion concentration supplied at different flow rates was evaluated (Figure S6). The response was found to be independent of the flow rate.

The operational and storage stability of the chloride ion sensor was also evaluated (Figures S7 and S8). The sensor was found to be sufficiently stable. These results suggest that the fabricated sensor can be used for real-time measurements.

### Selectivity of the Sensor

Because human sweat contains various ions, selectivity is an important parameter for evaluating the sensor response. The similar responses obtained with chloride solution and artificial sweat containing chloride (Figures S2 and 5, respectively) indicate that NH_4_^+^, SO_4_^2–^, K^+^, NO_3_^–^, Mg^2+^, glucose, and urea at physiological concentrations do not influence the sensor response. Nevertheless, the selectivity coefficients (log *K*_Cl^–^_,_NO_3_^–^_, log*K*_Cl^–^_,_SO_4_^2–^_) of the chloride ion sensor for two anions (NO_3_^–^ and SO_4_^2–^) abundantly contained in sweat were calculated using the separate solution method (Table 1). For this, the sensor response to each anion was evaluated, and the sensitivity was compared to that of the chloride ion.

**Table 1:** Selectivity Coefficients of the Chloride-Ion-Selective Membrane

analyte (Y)	log*K*_Cl^–^,Y_
NO_3_^–^	–1.1
SO_4_^2–^	–1.8

Typical values of NO_3_^–^ in sweat range from 0.5 to 4 mM,^43,44^ SO_4_^–^ range from 0 to 5 mM,^43^ and Cl^–^ range from 10 to 100 mM.^18,47^ The selectivity coefficients and typical concentration ranges suggest that any interference caused by these ions does not significantly affect the monitoring of chloride ions in human sweat using the fabricated ion sensor.

Additionally, selectivity coefficients of the potassium and sodium ion sensors for some of the cations were determined (Table S1). The selectivity coefficients and typical concentration ranges suggest that any interference caused by these ions does not significantly affect the monitoring of potassium and sodium ions in human sweat using the fabricated ion sensor.

Furthermore, the chloride ion sensor showed a stable response independent of the pH of the measurement solution (Figure S9), indicating that the pH of human sweat should not influence the sensor.

### On-Body Testing

**Figure 7:**
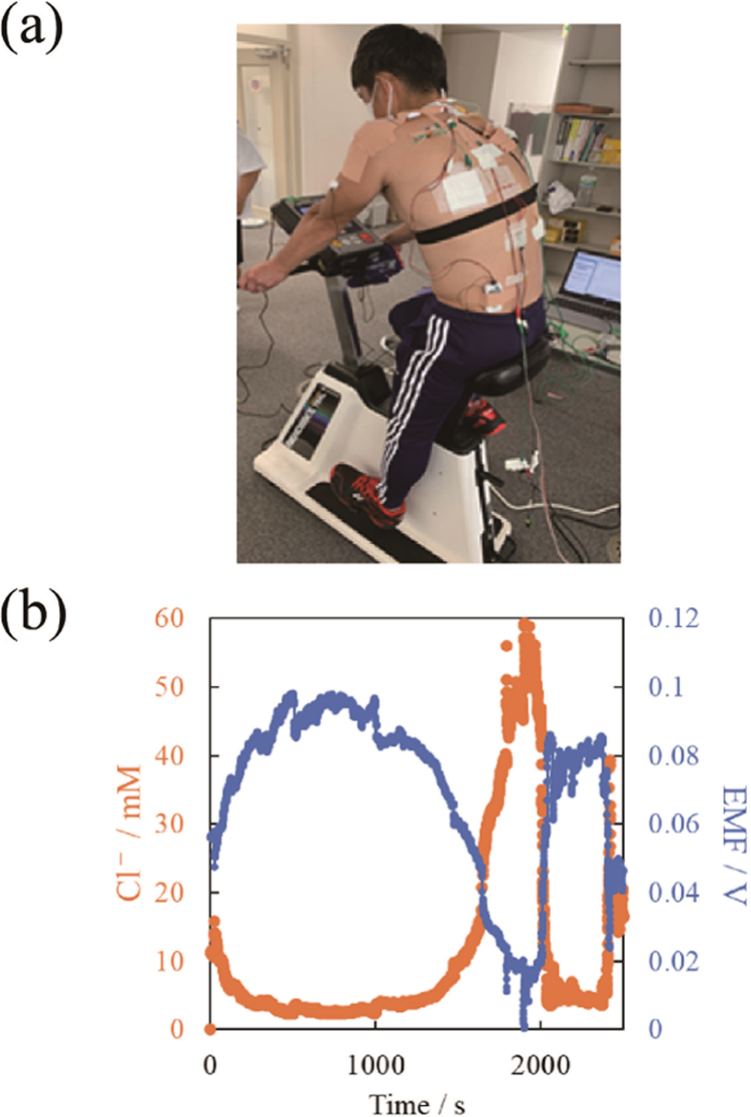
On-body test. (a) Photograph of a test subject with sensors taped to his body. (b) Response of chloride ion sensor during the on-body test.

**Figure 8:**
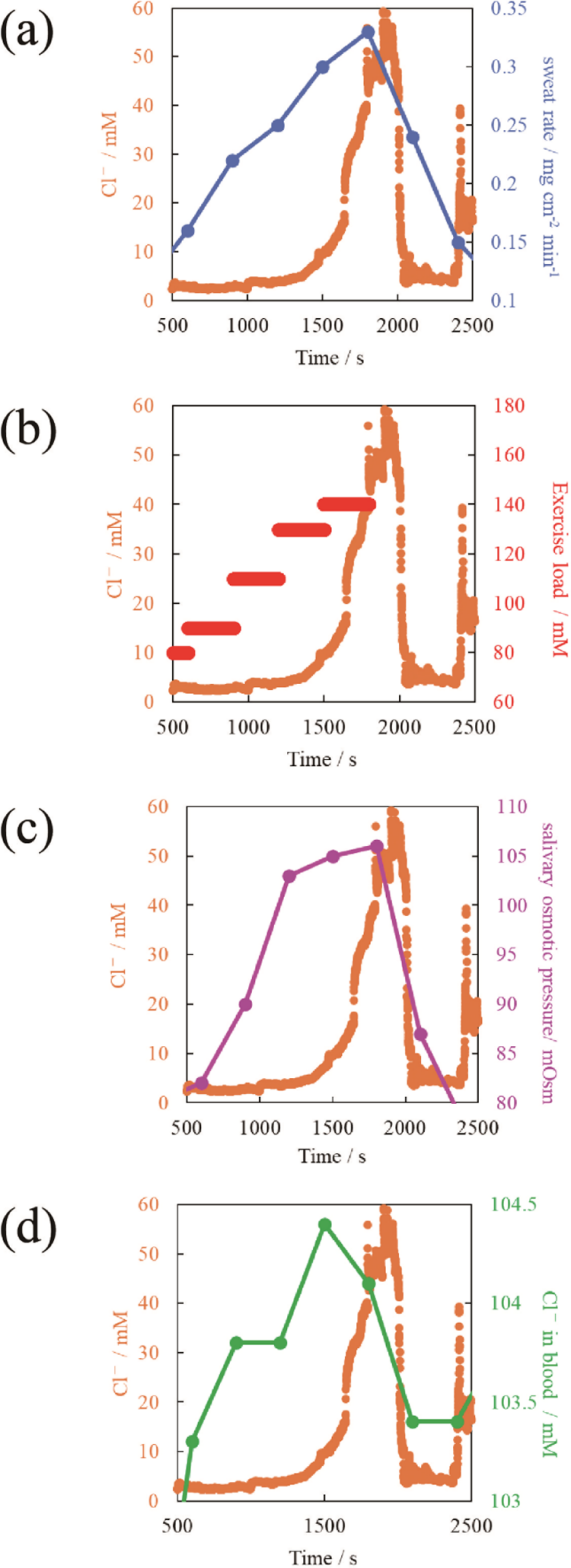
Correlation between sweat chloride ion concentration and other parameters. (a) Sweat rate, (b) exercise load, (c) degree of dehydration, and (d) chloride ion concentration in blood.

An on-body test was conducted using a fabricated transfer-printed chloride ion sensor. To continuously remove the old sweat from the sensing part of the sensor, a SAF was attached to the sensor cloth, creating a sweat flow path. The mechanism of continuous water absorption by the SAF is shown in Figure S9. The sensor was taped to the body of a healthy 40-year-old male exercising on an aerobic bike. The aerobic cycling exercise had a total duration of 30 min with a gradually increasing exercise load. Perspiration rate, chloride ion concentration in blood, and saliva osmolality (an indicator of dehydration level) were measured at 5 min intervals using commercial sensors. Figure 7a shows the on-body test in progress. Figure 7b shows the response of the chloride ion sensor during the on-body test. Chloride ion concentrations were calculated using the calibration curve shown in Figure 5. The open-circuit potential of the chloride ion sensor gradually decreased once sweating was observed (at about 800 s, Figure 7b). After the end of the exercise during the resting period, the open-circuit potential of the chloride ion sensor increased. Therefore, the chloride ion concentration in sweat increased with increasing exercise time and load and decreased after exercise. This trend is similar to those observed and reported in the literature.^47,48^ The correlations between the concentration of chloride ions in sweat measured in the on-body test and other physiological parameters are shown in Figure 8. All parameters showed close correlations with the chloride ion concentration in the measured sweat. In particular, the close correlation with saliva osmolality, which is an indicator of the degree of dehydration, suggests that the response of the chloride ion sensor developed in this study can be considered an indicator of the degree of dehydration.

### Physiological Background

Before sweat reaches the skin surface, electrolytes in the primary sweat are reabsorbed in the sweat duct to maintain electrolyte balance.^49,50^ Chloride ions are reabsorbed by cystic fibrosis transmembrane regulator (CFTR) channels in an adenosine triphosphate (ATP)-dependent mechanism.^49,51^ The chloride concentration in the final sweat thus depends on the ratio between the primary sweat production rate and the chloride ion reabsorption rate. Increased sweating and/or decreased reabsorption through the CFTR channels might have led to the increased chloride ion concentration in sweat observed during the increased exercise intensity in this study.

## Conclusions

In this study, we successfully developed a wearable sensor for continuously monitoring chloride ions in sweat. Screen-printed ion sensors were fabricated using heat-transfer printing. The chloride ion sensor showed a Nernst response of −59.5 ± 3.4 mV/log *C*_Cl^–^_ in the 0.1–100 mM concentration range. The ion sensor was fabricated with a soft, skin-friendly, nonwoven cloth. Therefore, the ion sensor should be comfortable to wear for extended periods without causing skin irritation. In the on-body test, chloride ion concentrations in sweat increased with exercise time, which is in agreement with previously reported results. Thus, the developed ion sensors have great potential for the practical application of the continuous monitoring of sweat ions, an important chemical parameter associated with physical training and exercise.

## Supporting Information Available

Additional experimental details, materials and methods, including additional characterization of the chloride ion sensor, as well as fabrication and characterization of sodium, potassium, and ammonium ion sensors (Figures S1–S10) and additional selectivity coefficients (Table S1) (PDF)

## Abbreviations

ATP: adenosine triphosphate
CFTR: cystic fibrosis transmembrane regulator
ATP: adenosine triphosphate
EMF: electromotive force
*o*-NPOE: *o*-nitrophenyl octyl ether
PET: polyester
PVB: poly(vinyl butyral) BUTVAR B-98
PVC: poly(vinyl chloride)
RE: reference electrode
SAF: superabsorbent fibers
TDDMACl: tridodecyl(methyl)ammonium chloride
THF: tetrahydrofuran
WE: working electrode

## References

[ref1] Fujii K. Study on the transmission mechanism for wearable device using the human body as a transmission channel. IEICE Trans. Commun. 2005, E88–B, 2401–2410. 10.1093/ietcom/e88-b.6.2401.

[ref2] Windmiller J. R.; Wang J. Wearable electrochemical sensors and biosensors: A review. Electroanalysis 2013, 25, 29–46. 10.1002/elan.201200349.

[ref3] Dubal D. P.; Chodankar N. R.; Kim D. H.; Gomez-Romero P. Towards flexible solid-state supercapacitors for smart and wearable electronics. Chem. Soc. Rev. 2018, 47, 2065–2129. 10.1039/C7CS00505A. 29399689

[ref4] Gao W.; Emaminejad S.; Nyein H. Y. Y.; Challa S.; Chen K. V.; Peck A.; Fahad H. M.; Ota H.; Shiraki H.; Kiriya D.; Lien D. H.; Brooks G. A.; Davis R. W.; Javey A. Fully integrated wearable sensor arrays for multiplexed in situ perspiration analysis. Nature 2016, 529, 509–514. 10.1038/nature16521. 26819044 PMC4996079

[ref5] Koh A.; Kang D.; Xue Y.; Lee S.; Pielak R. M.; Kim J.; Hwang T.; Min S.; Banks A.; Bastien P.; Manco M. C.; Wang L.; Ammann K. R.; Jang K. I.; Won P.; Han S.; Ghaffari R.; Paik U.; Slepian M. J.; Rogers J. A.; et al. A soft, wearable microfluidic device for the capture, storage, and colorimetric sensing of sweat. Sci. Trans. Med. 2016, 8, 336ra165 10.1126/scitranslmed.aaf2593. PMC5429097 27881826

[ref6] Bhattacharya D.; Bhattacharjee P. “Epidermal Electronics” printed on the membrane AS tattoo. Int. J. Innovative Res. Dev. 2013, 2, 437–443.

[ref7] Yeo W. H.; Kim Y. S.; Lee J.; Ameen A.; Shi L.; Li M.; Wang S.; Ma R.; Jin S. H.; Kang Z.; Huang Y.; Rogers J. A. Multifunctional epidermal electronics printed directly onto the skin. Adv. Mater. 2013, 25, 2773–2778. 10.1002/adma.201204426. 23440975

[ref8] Jia W.; Bandodkar A. J.; Valdés-Ramírez G.; Windmiller J. R.; Yang Zhanjun.; Ramírez J.; Chan G.; Wang J. Electrochemical tattoo biosensors for real-time noninvasive lactate monitoring in human perspiration. Anal. Chem. 2013, 85, 6553–6560. 10.1021/ac401573r. 23815621

[ref9] Coyle S.; Lau K.-T.; Moyna N.; O’Gorman D.; Diamond D.; Di Francesco F.; Costanzo D.; Salvo P.; Trivella M. G.; De Rossi D. E.; Taccini N.; Paradiso R.; Porchet J.-A.; Ridolfi A.; Luprano J.; Chuzel C.; Lanier T.; Revol-Cavalier F.; Schoumacker S.; Mourier V.; Chartier I.; Convert R.; De-Moncuit H.; Bini C. BIOTEX—Biosensing Textiles for Personalised Healthcare Management, IEEE Transactions on Information Technology in Biomedicine, 2010; pp 364–370. 10.1109/TITB.2009.2038484 20064761

[ref10] Chu M. X.; Miyajima K.; Takahashi D.; Arakawa T.; Sano K.; Sawada S.; Kudo H.; Iwasaki Y.; Akiyoshi K.; Mochizuki M.; Mitsubayashi K. Soft contact lens biosensor for in situ monitoring of tear glucose as non-invasive blood sugar assessment. Talanta 2011, 83, 960–965. 10.1016/j.talanta.2010.10.055. 21147344

[ref11] Shitanda I.; Nohara S.; Hoshi Y.; Itagaki M.; Tsujimura S. A screen-printed circular-type paper-based glucose/O_2_ biofuel cell. J. Power Sources 2017, 360, 516–519. 10.1016/j.jpowsour.2017.06.043.

[ref12] Shitanda I.; Momiyama M.; Watanabe N.; Tanaka T.; Tsujimura S.; Hoshi Y.; Itagaki M. Toward wearable energy storage devices: Paper-based biofuel cells based on a screen-printing array structure. ChemElectroChem 2017, 4, 2460–2463. 10.1002/celc.201700561. 29214125 PMC5708273

[ref13] Xu G.; Cheng C.; Yuan W.; Liu Z. Y.; Zhu L. H.; Li X. T.; Lu Y. L.; Chen Z. T.; Liu J. L.; Cui Z.; Kang Z. H.; Zhang H. Y.; Ma Y. W.; Sui X. Y.; Tao X. Y.; Han H. B.; Li X. Y.; Li T.; Sun J. Y. Smartphone-based battery-free and flexible electrochemical patch for calcium and chloride ions detections in biofluids. Sens. Actuators, B 2019, 297, 126743 10.1016/j.snb.2019.126743.

[ref14] Possanzini L.; Decataldo F.; Mariani F.; Gualandi I.; Tessarolo M.; Scavetta E.; Fraboni B. Textile sensors platform for the selective and simultaneous detection of chloride ion and pH in sweat. Sci. Rep. 2020, 10, 17180 10.1038/s41598-020-74337-w. 33057081 PMC7560666

[ref15] Nyein H. Y. Y.; Bariya M.; Tran B.; Ahn C. H.; Brown B. J.; Ji W. B.; Davis N.; Javey A. A wearable patch for continuous analysis of thermoregulatory sweat at rest. Nat. Commun. 2021, 12, 1823 10.1038/s41467-021-22109-z. 33758197 PMC7987967

[ref16] Choi D. H.; Gonzales M.; Kitchen G. B.; Phan D. T.; Searson P. C. A capacitive sweat rate sensor for continuous and real-time monitoring of sweat loss. ACS Sens. 2020, 5, 3821–3826. 10.1021/acssensors.0c01219. 33263987

[ref17] Kim S. B.; Zhang Y.; Won S. M.; Bandodkar A. J.; Sekine Y.; Xue Y. G.; Koo J.; Harshman S. W.; Martin J. A.; Park J. M.; Ray T. R.; Crawford K. E.; Lee K.-T.; Choi J.; Pitsch R. L.; Chung H. U.; Lee S.-P.; Yang Y.; Byun J.-H.; Lee S.; Luo S.; Koh J.; Qu M.; Lee U.; Chung J.; Kim J.; Lee J.; Shim H. J.; Han S.; Park I.; Hwang B. H.; Kwon K. Y.; Yeo W. H.; Huang Y.; Montana M. C.; Song J.; Ha J. S.; Martin B. E.; Yin L.; Cheng H.; Kang S. K.; Gerold B.; Slepian M.; Huang Y.; Armand M.; Choi W. K.; Bretl T.; Choi H.; Xu S.; Kamper D.; Omenetto F.; Huang Y.; Ray W. Z.; Rogers J. A. Super-absorbent polymer valves and colorimetric chemistries for time-sequenced discrete sampling and chloride analysis of sweat via skin-mounted soft microfluidics. Small 2018, 14, 1703334 10.1002/smll.201703334. 29394467

[ref18] Parrilla M.; Cuartero M.; Crespo G. A. Wearable potentiometric ion sensors. TrAC, Trends Anal. Chem. 2019, 110, 303–320. 10.1016/j.trac.2018.11.024.

[ref19] Xu J. N.; Zhang Z.; Gan S. Y.; Gao H.; Kong H. J.; Song Z. Q.; Ge X. M.; Bao Y.; Niu L. Highly stretchable fiber-based potentiometric ion sensors for multichannel real-time analysis of human sweat. ACS Sen. 2020, 5, 2834–2842. 10.1021/acssensors.0c00960. 32854495

[ref20] Luetkemeier M. J.; Coles M. G.; Askew E. W. Dietary sodium and plasma volume levels with exercise. Sports Med. 1997, 23, 279–286. 10.2165/00007256-199723050-00002. 9181666

[ref21] Rosner M. H.; Kirven J.; Clin J. Exercise-associated hyponatremia. Am. Soc. Nephrol. 2007, 2, 151–161. 10.2215/CJN.02730806. 17699400

[ref22] Farrell P. M.; Rosenstein B. J.; White T. B.; Accurso F. J.; Castellani C.; Cutting G. R.; Durie P. R.; LeGrys V. A.; Massie J.; Parad R. B.; Rock M. J.; Campbell P. W. Jr. Guidelines for diagnosis of cystic fibrosis in newborns through older adults: Cystic Fibrosis Foundation consensus report. J. Pediatr. 2008, 153, S4–S14. 10.1016/j.jpeds.2008.05.005. 18639722 PMC2810958

[ref23] Ramsey B. W.; Davies J.; McElvaney N. G.; Tullis E.; Bell S. C.; Drevinek P.; Griese M.; McKone E. F.; Wainwright C. E.; Konstan M. W.; Moss R.; Ratjen F.; Sermet-Gaudelus I.; Rowe S. M.; Dong Q.; Rodriguez S.; Yen K.; Ordonez C.; Elborn J. S. A CFTR potentiator in patients with cystic fibrosis and the G551D mutation. N. Engl. J. Med. 2011, 365, 1663–1672. 10.1056/NEJMoa1105185. 22047557 PMC3230303

[ref24] Guinovartab T.; Bandodkara A. J.; Windmillerac J. R.; Andrade F. J.; Wang J. A potentiometric tattoo sensor for monitoring ammonium in sweat. Anal. Chem. 2013, 138, 7031–7038. 10.1039/C3AN01672B. 24098883

[ref25] Choi D. H.; Kim J. S.; Cutting G. R.; Searson P. C. Wearable potentiometric chloride sweat sensor: the critical role of the salt bridge. Anal. Chem. 2016, 88, 12241–12247. 10.1021/acs.analchem.6b03391. 28193033

[ref26] Cazalé A.; Sant W.; Ginot F.; Launay J.-C.; Savourey G.; Revol-Cavalier F.; Lagarde J. M.; Heinry D.; Launay J.; Temple-Boyer P. Physiological stress monitoring using sodium ion potentiometric microsensors for sweat analysis. Sens. Actuators, B 2016, 225, 1–9. 10.1016/j.snb.2015.10.114.

[ref27] Wujcik E. K.; Blasdel N. J.; Trowbridge D.; Monty C. N. Ion sensor for the quantification of sodium in sweat samples. IEEE Sens. J. 2013, 13, 3430–3436. 10.1109/JSEN.2013.2257168.

[ref28] Glennon T.; O’Quigley C.; McCaul M.; Matzeu G.; Beirne S.; Wallace G. G.; Stroiescu F.; O’Mahoney N.; White P.; Diamond D. ‘SWEATCH’: A wearable platform for harvesting and analysing sweat sodium content. Electroanalysis 2016, 28, 1283–1289. 10.1002/elan.201600106.

[ref29] Parrilla M.; Ferré J.; Guinovart T.; Andrade F. J. Wearable potentiometric sensors based on commercial carbon fibres for monitoring sodium in sweat. Electroanalysis 2016, 28, 1267–1275. 10.1002/elan.201600070.

[ref30] Matzeu G.; O’Quigley C.; McNamara E.; Zuliani C.; Fay C.; Glennon T.; Diamond D. An integrated sensing and wireless communications platform for sensing sodium in sweat. Anal. Methods 2016, 8, 64–71. 10.1039/C5AY02254A.

[ref31] Rose D. P.; Ratterman M. E.; Griffin D. K.; Hou L.; Kelley-Loughnane N.; Naik R. R.; Hagen J. A.; Papautsky I.; Heikenfeld J. C. Adhesive RFID sensor patch for monitoring of sweat electrolytes. IEEE Trans. Biomed. Eng. 2015, 62, 1457–1465. 10.1109/TBME.2014.2369991. 25398174

[ref32] Schazmann B.; Morris D.; Slater C.; Beirne S.; Fay C.; Reuveny R.; Moyna N.; Diamond D. A wearable electrochemical sensor for the real-time measurement of sweat sodium concentration. Anal. Methods 2010, 2, 342–348. 10.1039/b9ay00184k.

[ref33] Zoerner A.; Oertel S.; Jank M. P. M.; Frey L.; Langenstein B.; Bertsch T. Human sweat analysis using a portable device based on a screen-printed electrolyte sensor. Electroanalysis 2018, 30, 665–671. 10.1002/elan.201700672.

[ref34] Ichimura Y.; Kuritsubo T.; Nagamine K.; Nomura A.; Shitanda I.; Tokito A fully screen-printed potentiometric chloride ion sensor employing a hydrogel-based touchpad for simple and non-invasive daily electrolyte analysis. Anal. Bioanal. Chem. 2021, 413, 1883–1891. 10.1007/s00216-021-03156-3. 33479820

[ref35] Bandodkar A. J.; Molinnus D.; Mirza O.; Guinovart T.; Windmiller J. R.; Valdés-Ramírez G.; Andrade F. J.; Schöning M. J.; Wang J. Epidermal tattoo potentiometric sodium sensors with wireless signal transduction for continuous non-invasive sweat monitoring. Biosens. Bioelectron. 2014, 54, 603–609. 10.1016/j.bios.2013.11.039. 24333582

[ref36] Malinowska E.; Niedziolka J.; Meyerhoff M. E. Potentiometric and spectroscopic characterization of anion selective electrodes based on metal(III) porphyrin ionophores in polyurethane membranes. Anal. Chim. Acta 2001, 432, 67–78. 10.1016/S0003-2670(00)01348-9.

[ref37] Wang E. J.; Meyerhoff M. E. Anion selective optical sensing with metalloporphyrin-doped polymeric films. Anal. Chim. Acta 1993, 283, 673–682. 10.1016/0003-2670(93)85281-N.

[ref38] Xiao K. P.; Buhlmann P.; Nishizawa S.; Amemiya S.; Umezawa Y. A chloride ion-selective solvent polymeric membrane electrode based on a hydrogen bond forming ionophore. Anal. Chem. 1997, 69, 1038–1044. 10.1021/ac961035d.

[ref39] Guinovart T.; Crespo G. A.; Rius F. X.; Andrade F. J. A reference electrode based on polyvinyl butyral (PVB) polymer for decentralized chemical measurements. Anal. Chim. Acta 2014, 821, 72–80. 10.1016/j.aca.2014.02.028. 24703216

[ref40] Paul B.; Demuru S.; Lafaye C.; Saubade M.; Briand D. Printed iontophoretic-integrated wearable microfluidic sweat-sensing patch for on-demand point-of-care sweat analysis. Adv. Mater. Technol. 2021, 6, 2000910 10.1002/admt.202000910.

[ref41] Xu G.; Cheng C.; Liu Z. Y.; Yuan W.; Wu X. Z.; Lu Y. L.; Low S. S.; Liu J. L.; Zhu L. H.; Ji D. Z.; et al. Battery-free and wireless epidermal electrochemical system with all-printed stretchable electrode array for multiplexed in situ sweat analysis. Adv. Mater. Technol. 2019, 4, 1800658 10.1002/admt.201800658.

[ref42] Nyein H. Y. Y.; Gao W.; Shahpar Z.; Emaminejad S.; Challa S.; Chen K.; Fahad H. M.; Tai L.-C.; Ota H.; Davis R. W.; Javey A. A wearable electrochemical platform for noninvasive simultaneous monitoring of Ca^2+^ and pH. ACS Nano 2016, 10, 7216–7224. 10.1021/acsnano.6b04005. 27380446

[ref43] Sato K.; Kang W. H.; Saga K.; Sato K. T. J. Biology of sweat glands and their disorders. I. Normal sweat gland function. Am. Acad. Dermatol. 1989, 20, 537–563. 10.1016/S0190-9622(89)70063-3. 2654204

[ref44] Patterson M. J.; Galloway S. D. R.; Nimmo M. A. Variations in regional sweat composition in normal human males. Exp. Physiol. 2000, 85, 869–875. 10.1111/j.1469-445X.2000.02058.x. 11187982

[ref47] Choi D. H.; Kitchen G. B.; Stewart K. J.; Searson P. C. The dynamic response of sweat chloride to changes in exercise load measured by a wearable sweat sensor. Sci. Rep. 2020, 10, 7699 10.1038/s41598-020-64406-5. 32382047 PMC7205967

[ref48] Choi D. H.; Li Y.; Cutting G. R.; Searson P. C. A wearable potentiometric sensor with integrated salt bridge for sweat chloride measurement. Sens. Actuators, B 2017, 250, 673–678. 10.1016/j.snb.2017.04.129.

[ref49] Sheppard D. N.; Welsh M. J. Structure and function of the CFTR chloride channel. Physiol. Rev. 1999, 79, S23–45. 10.1152/physrev.1999.79.1.S23. 9922375

[ref50] Baker L. B.; Wolfe A. S. Physiological mechanisms determining eccrine sweat composition. Eur. J. Appl. Physiol. 2020, 120, 719–752. 10.1007/s00421-020-04323-7. 32124007 PMC7125257

[ref51] Quinton P. M. Sweating and its disorders. Annu. Rev. Med. 1983, 34, 429–452. 10.1146/annurev.me.34.020183.002241. 6344770

