# Supplementary material for: Wearable Ion Sensors for the Detection of Sweat Ions Fabricated by Heat-Transfer Printing
Source: ACS Sens. 2023 Jun 15;8(7):2889–95. doi: 10.1021/acssensors.3c01027 (PMC10391709; doi:10.1021/acssensors.3c01027)
Supplement: Supplementary file 1 — se3c01027_si_001.pdf [file se3c01027_si_001.pdf]

**Supplemental Material**

# Wearable Ion Sensors for the Detection of Sweat Ions Fabricated by Heat-transfer Printing

Isao Shitanda,[1,2*] Naoki Muramatsu,[1‡] Rio Kimura,[1‡] Nanami Takahashi,[1‡] Kazuki Watanabe,[1‡] Hiroyuki Matsui,[3] Noya Loew,[1,‡] Masahiro Motosuke,[2,4] Takahiro Mukaimoto,[2,5] Momoko Kobayashi,[6] Taketo Mitsuhara,[7] Yamato Sugita,[7] Kensuke Matsuo,[7] Shinya Yanagita,[2,5] Tatsunori Suzuki,[6] Hikari Watanabe,[1] and Masayuki Itagaki[1,2]

[1] Department of Pure and Applied Chemistry, Faculty of Science and Technology, Tokyo University of Science, 2641 Yamazaki, Noda, Chiba 278-8510, Japan

[2] Research Institute for Science and Technology, Tokyo University of Science, 2641 Yamazaki, Noda, Chiba 278-8510, Japan

[3] Research Center for Organic Electronics (ROEL), Yamagata University, 4-3-16 Jonan, Yonezawa, Yamagata 992-8510, Japan

[4] Department of Mechanical Engineering, Faculty of Engineering, Tokyo University of Science, 6-3-1 Niijuku, Katsushika-ku, Tokyo 125-8585, Japan

[5] Institute of Arts and Sciences, Tokyo University of Science, 2641 Yamazaki, Noda, Chiba 278-8510, Japan

[6] Department of Pharmacy, Faculty of Pharmaceutical Sciences, Tokyo University of Science, 2641 Yamazaki, Noda, Chiba 278-8510, Japan

[7] Department of Globe Fire Science and Technology, Faculty of Science and Technology, Tokyo University of Science, 2641 Yamazaki, Noda, Chiba 278-8510, Japan

‡ These authors contributed equally

[*]Corresponding author: shitanda@rs.tus.ac.jp

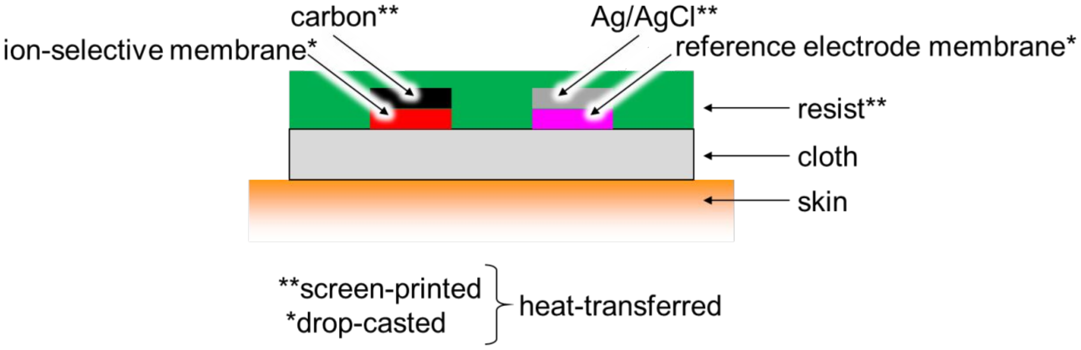


**Figure S1. Schematic of the heat-transfer printed ion sensor on skin.** Cross-section through the sensing electrodes. Sensor component layers are formed on PET film by screen-printing or drop-casting as indicated, and then heat-transferred onto cloth. The cloth is in direct contact with the skin during use, while the sensor component layers face away from the skin.

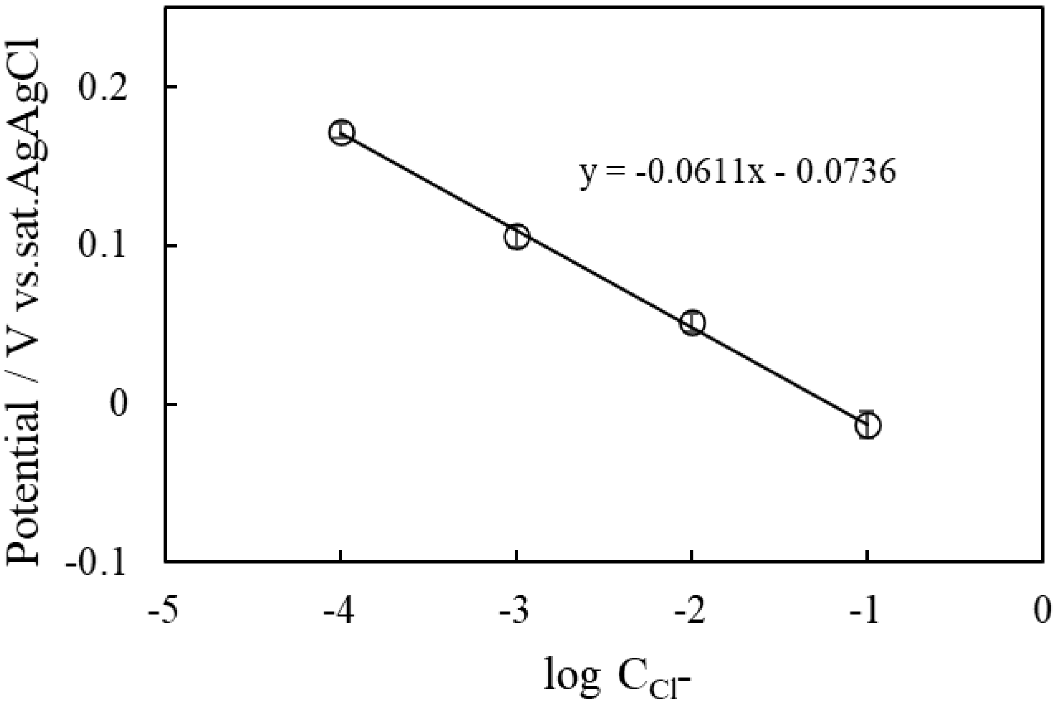


**Figure S2. Chloride ion electrode.** The potential of the chloride ion electrode in the presence of various chloride ion concentrations was measured against a commercial reference electrode. The value at 60 s after the start of the measurement was plotted against the chloride ion concentration.

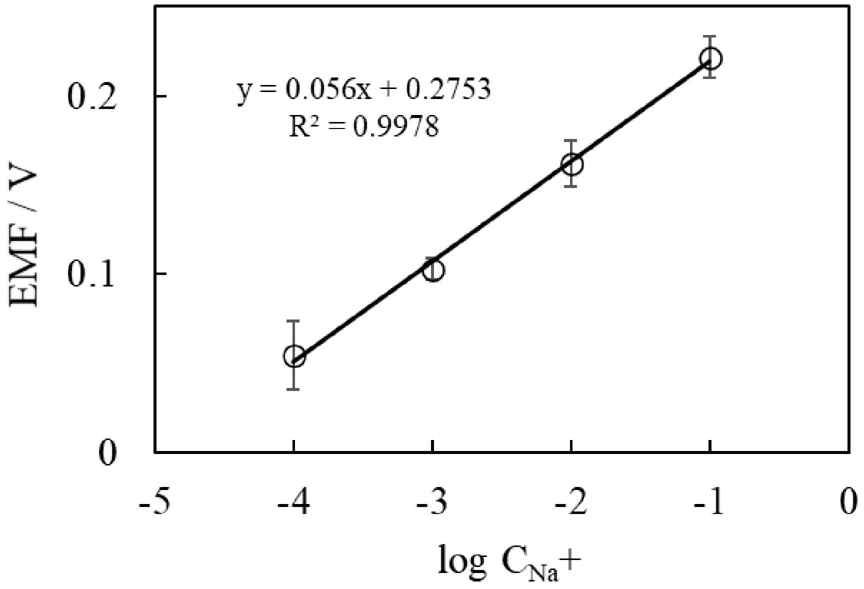


**Figure S3. Sodium ion sensor.**

**Sodium ion sensor fabrication**

For the sodium ion sensor, a mixture of 2 mg sodium ionophore X, 1 mg Tetrakis[3,5-bis(trifluoromethyl)phenyl]borate, sodium salt (Na-TFPB), 33 mg PVC, and 65.5 mg dioctyl sebacate (DOS) was dissolved in 700 µL of THF. Then, 8 µL of this mixture was dropped onto a carbon electrode and dried overnight to form a sodium ion-selective membrane. The reference electrode membrane was prepared similarly to that for the chloride ion sensor but without Pluronic F-127.

**Sodium ion sensor characterization**

The sodium ion sensor was characterized by measuring the sensor response to various concentrations of sodium chloride in artificial sweat. The dependency of sensor EMF on the sodium concentration is shown in Fig. S3 (above).

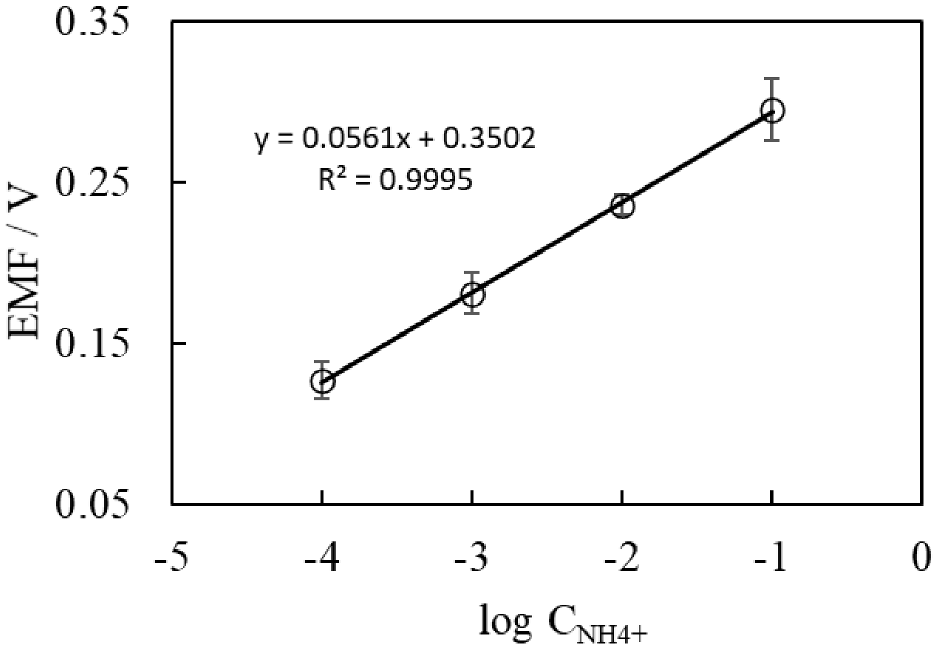


**Figure S4. Ammonium ion sensor.**

**Ammonium ion sensor fabrication**

For the ammonium ion sensor, a mixture of 4 mg ammonium ionophore I, 1 mg Tetrakis[3,5-bis(trichloromethyl)phenyl]borate, potassium salt (KTCIPB), 130 mg PVC, and 260 mg o-NPOE was dissolved in 1.0 mL of THF. Then, 8 μL of this mixture was dropped onto a carbon electrode and dried overnight to form an ammonium ion-selective membrane. The reference electrode membrane was prepared similarly to that for the chloride ion sensor but without Pluronic F-127.

**Ammonium ion sensor characterization**

The ammonium ion sensor was characterized by measuring the sensor response to various concentrations of ammonium chloride in artificial sweat. The dependency of sensor EMF on the ammonium concentration is shown in Fig. S4 (above).

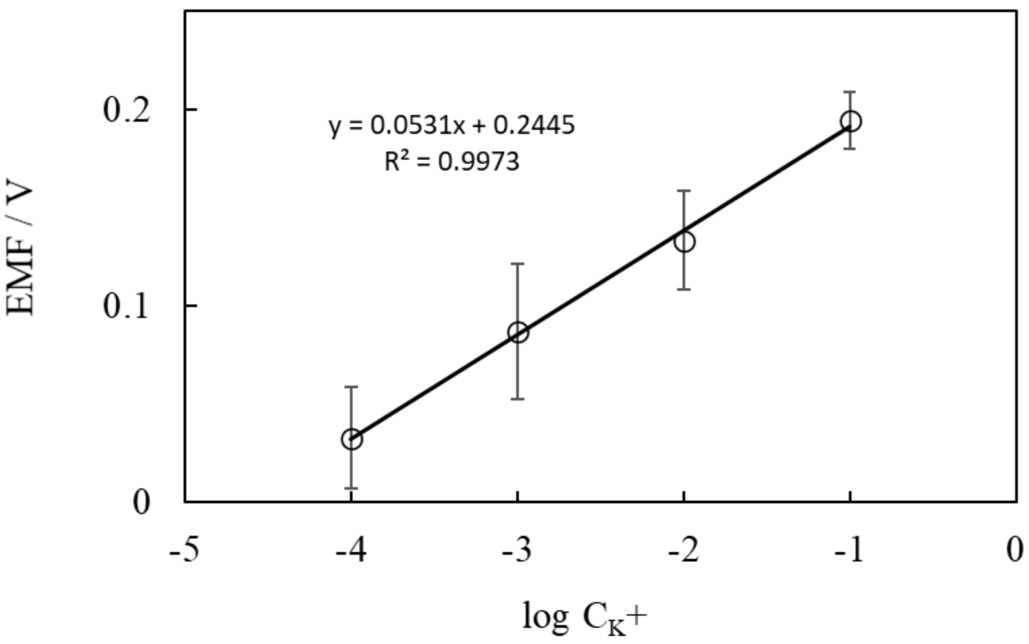


**Figure S5. Potassium ion sensor.**

**Potassium ion sensor fabrication**

For the potassium ion sensor, a mixture of 2 mg valinomycin, 0.7 mg KTCIPB, 33 mg PVC, and 65.45 mg o-NPOE was dissolved in 660 μL of THF. Then, 8 μL of this mixture was dropped onto a carbon electrode and dried overnight to form a potassium ion-selective membrane. The reference electrode membrane was prepared similarly to that for the chloride ion sensor but without Pluronic F-127.

**Potassium ion sensor characterization**

The potassium ion sensor was characterized by measuring the sensor response to various concentrations of potassium chloride in artificial sweat. The dependency of sensor EMF on the potassium concentration is shown in Fig. S5 (above).

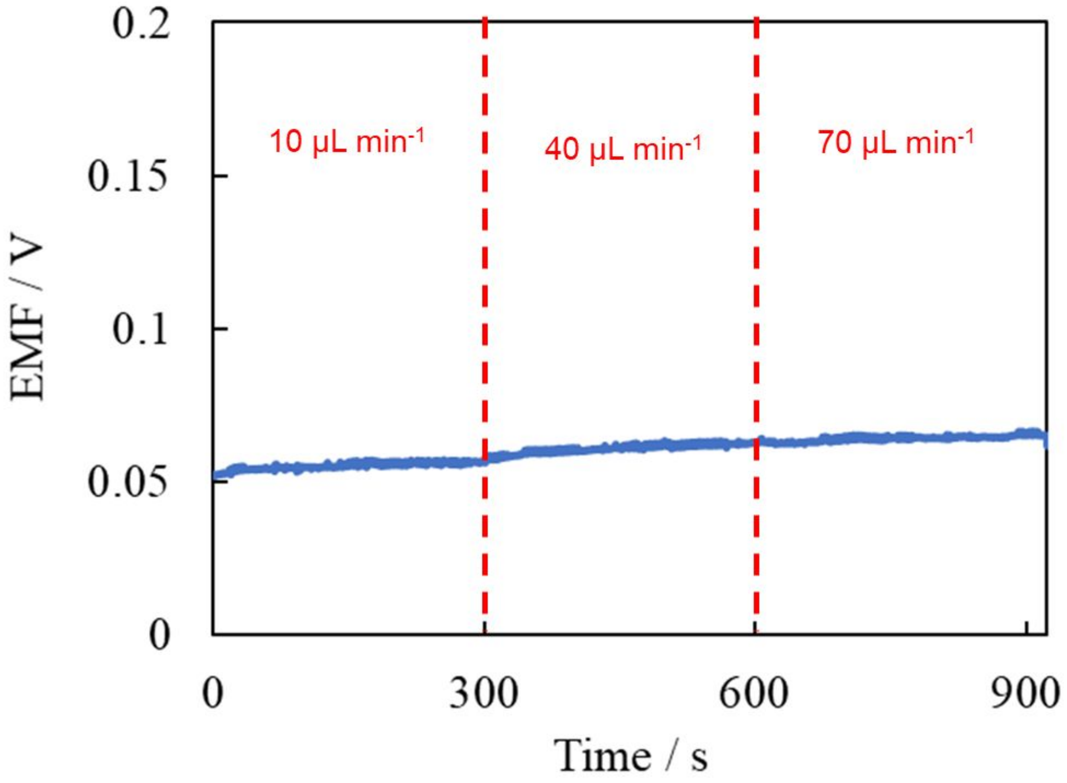


**Figure S6. Flow rate dependency.** A 10 mM sodium chloride solution was applied to the chloride ion sensor using a syringe pump, and the response was recorded. The flow rate was increased at 300 s intervals from 10 to 40 μL $min^{-2}$, and finally to 70 μL $min^{-2}$.

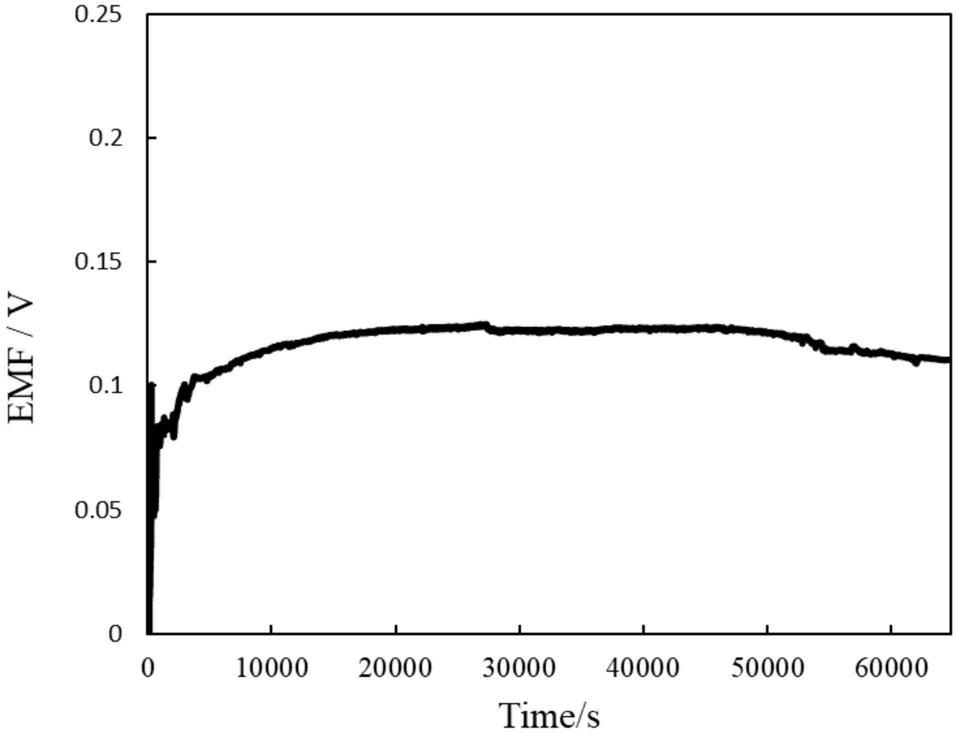


**Figure S7. Operational stability.** The operational stability of the chloride ion sensor was evaluated by measuring the EMF in the presence of 100 mM sodium chloride over 18 h. The sensor was found to be sufficiently stable during this time.

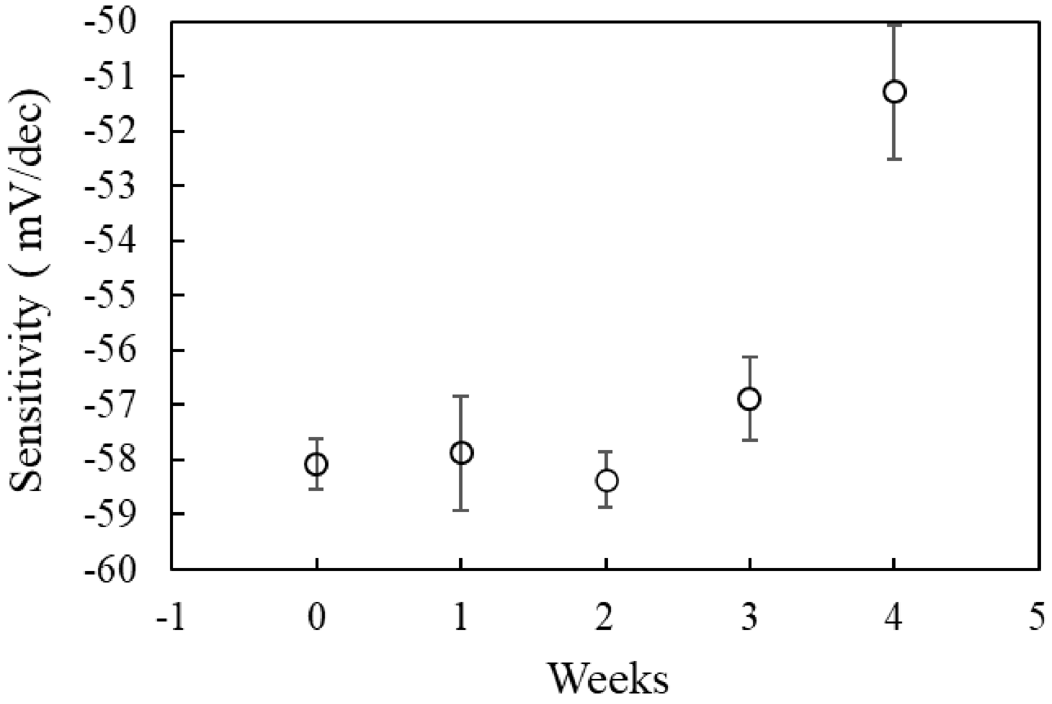


**Figure S8. Storage stability.** Chloride ion sensors were stored under ambient conditions for up to 4 weeks. The sensitivity of the sensors remained stable for 2 to 3 weeks (n = 3).

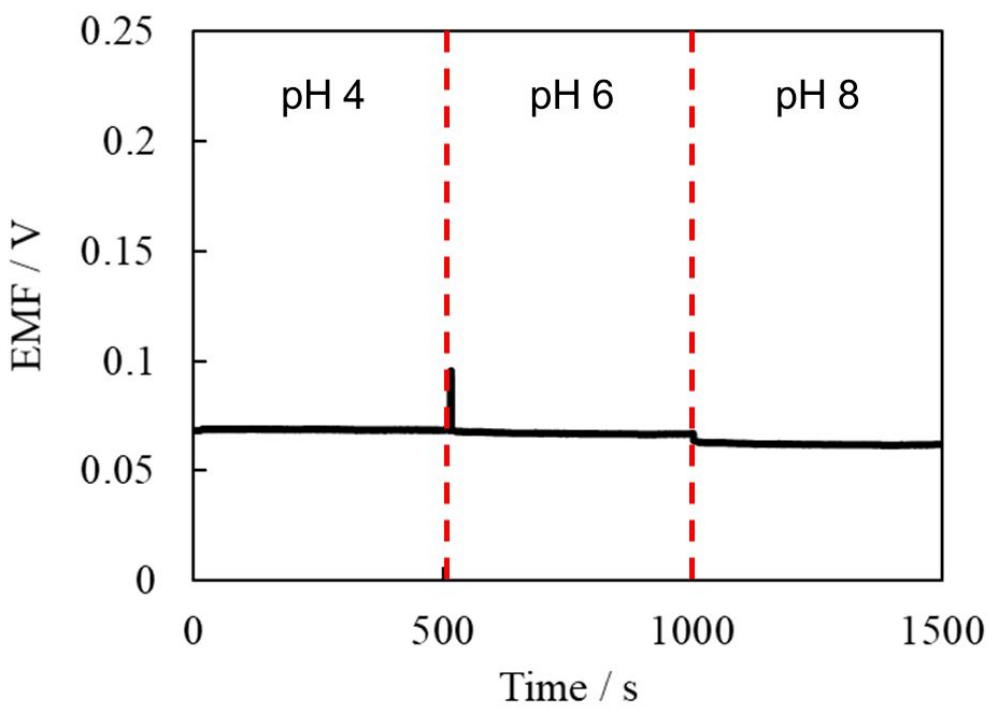


**Figure S9. pH dependency.** Response of the chloride ion sensor to 40 mM sodium chloride in McIlvaine buffer with different pH values. No dependency of the sensor response to pH was observed in the pH range of 4–8.

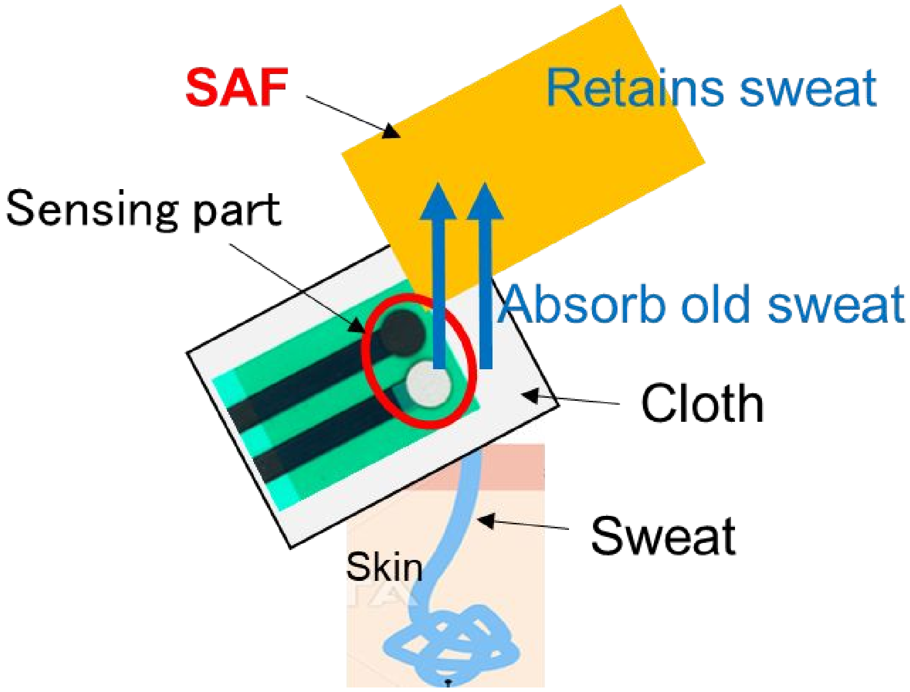


**Figure S10. Scheme of the sensor with SAF.** Sweat exuded from a sweat gland is collected by the cloth substrate of the sensor, and the ion concentration is measured. This old sweat is then absorbed by the SAF. Owing to the high water absorption capability of the SAF, it was capable of retaining and absorbing all the sweat during on-body testing.

**Table S1. Selectivity coefficients of potassium- and sodium-selective membranes.** Typical values in human sweat are: $K^+$ of 0.2–6 mM[1,2], $Na^+$ of 10–100 mM[1,2], $NH_4^+$ of 0.1–1 mM[1,3], and $Ca^{2+}$ of 0.5–3 mM[4]. The selectivity coefficients and typical concentration ranges suggest that any interference caused by these ions does not significantly affect the monitoring of potassium and sodium ions in human sweat using the respective fabricated ion sensors.

| Analyte X (selective ion) | Analyte Y | log $K_{X,Y}$ |
|---|---|---|
| $K^+$ | $Na^+$ | -2.7 |
| $K^+$ | $NH_4^+$ | -3.0 |
| $Na^+$ | $K^+$ | -2.9 |
| $Na^+$ | $Ca^{2+}$ | -4.3 |
| $Na^+$ | $NH_4^+$ | -3.9 |

*References*


1) Sato, K.; Kang, W. H.; Saga, K.; Sato, K. T. Biology of Sweat Glands and Their Disorders. I. Normal Sweat Gland Function. *J. Am. Acad. Dermatol.* **1989,** *20* (4), 537–563. https://doi.org/10.1016/S0190-9622(89)70063-3.
2) Patterson, M. J.; Galloway, S. D. R.; Nimmo, M. A. Variations in Regional Sweat Composition in Normal Human Males. *Exp. Physiol.* **2000,** *85* (6), 869–875. https://doi.org/10.1111/j.1469-445X.2000.02058.x.
3) Czarnowski, D.; Górski, J.; Jóźwiuk, J.; Boroń-Kaczmarska, A. Plasma Ammonia Is the Principal Source of Ammonia in Sweat. *Europ. J. Appl. Physiol.* **1992**, 65 (2), 135–137. https://doi.org/10.1007/BF00705070.
4) Nyein, H. Y. Y.; Gao, W.; Shahpar, Ziba; Emaminejad, S.; Challa, S.; Chen, K.; Fahad, H. M.; Tai, L.-C.; Ota, H.; Davis, R. W.; Javey, A. A Wearable Electrochemical Platform for Noninvasive Simultaneous Monitoring of $Ca^{2+}$ and pH. *ACS Nano* **2016,** *10* (7), 7216–7224. https://doi.org/10.1021/acsnano.6b04005.
